# The Detection of Orientation Continuity and Discontinuity by Cat V1 Neurons

**DOI:** 10.1371/journal.pone.0079723

**Published:** 2013-11-21

**Authors:** Tao Xu, Ling Wang, Xue-Mei Song, Chao-Yi Li

**Affiliations:** 1 Key Laboratory for Neuroinformation of Ministry of Education, University of Electronic Science and Technology of China, Chengdu, China; 2 Shanghai Institutes of Biological Sciences, Chinese Academy of Sciences, Shanghai, China; CSIC-Univ Miguel Hernandez, Spain

## Abstract

The orientation tuning properties of the non-classical receptive field (nCRF or “surround”) relative to that of the classical receptive field (CRF or “center”) were tested for 119 neurons in the cat primary visual cortex (V1). The stimuli were concentric sinusoidal gratings generated on a computer screen with the center grating presented at an optimal orientation to stimulate the CRF and the surround grating with variable orientations stimulating the nCRF. Based on the presence or absence of surround suppression, measured by the suppression index at the optimal orientation of the cells, we subdivided the neurons into two categories: surround-suppressive (SS) cells and surround-non-suppressive (SN) cells. When stimulated with an optimally oriented grating centered at CRF, the SS cells showed increasing surround suppression when the stimulus grating was expanded beyond the boundary of the CRF, whereas for the SN cells, expanding the stimulus grating beyond the CRF caused no suppression of the center response. For the SS cells, strength of surround suppression was dependent on the relative orientation between CRF and nCRF: an iso-orientation grating over center and surround at the optimal orientation evoked strongest suppression and a surround grating orthogonal to the optimal center grating evoked the weakest or no suppression. By contrast, the SN cells showed slightly increased responses to an iso-orientation stimulus and weak suppression to orthogonal surround gratings. This iso-/orthogonal orientation selectivity between center and surround was analyzed in 22 SN and 97 SS cells, and for the two types of cells, the different center-surround orientation selectivity was dependent on the suppressive strength of the cells. We conclude that SN cells are suitable to detect orientation continuity or similarity between CRF and nCRF, whereas the SS cells are adapted to the detection of discontinuity or differences in orientation between CRF and nCRF.

## Introduction

The responses of V1 neurons to visual stimuli presented in the classical receptive field (CRF or center) can be facilitated or suppressed by stimuli in the non-classical-receptive field (nCRF or surround) [1]–[14]. The center responses of most V1 cells are suppressed by surround stimuli [14]–[16] with maximal suppressive modulation occurring when the center and surround are stimulated at the identical orientation, drifting direction and spatiotemporal frequency [7], [9], [11], [17], [18]. Suppression is weaker if the surrounding orientation deviates from the optimal central grating, particularly when the orientation in the surround is 90° from the preferred orientation (orthogonal) [15], [19]. This effect may be linked to figure/ground segregation [7], [10], [20]. For some cells, facilitatory effects have also been observed when the surround stimuli match the central optimal orientation [21]–[23] and are eliminated by orthogonal surround stimuli [21], [23].

Previous studies [11], [19] of the primary visual cortex of anaesthetized primates revealed that surround orientation tuning depends on the contrast of the central stimulus. Combining intrinsic signal optical imaging and single-unit recording in the V1 of anesthetized cats, a recent study [24] reported that surround orientation selectivity might also depended on the location of the neurons in the optical orientation map of V1.

In the experiments presented here, we recorded single-unit activities from the V1 of anesthetized cats and determined the CRF size and suppression index (SI) of the neurons using a size-tuning test. In the experiment, a high-contrast grating, which was drifting at the optimal orientation/direction of the neuron, was varied in size, and the SI of the neuron was measured from the size-tuning curve. We classified the recorded neurons into two categories according to their SI, the “surround-non-suppressive” (SN, 0
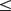
SI
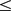
0.1) and the “surround-suppressive” (SS, 0.1
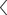
SI
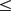
1).

We then performed surround orientation tuning tests. In these tests, the nCRF was stimulated by a larger circular grating (20°
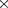
20°) that drifted randomly at different orientations/directions while a smaller optimally oriented grating was drifted within the CRF. We observed that the surround suppression was stronger at an iso-compared to ortho-orientation for SS neurons and vice versa for SN cells. These results may imply that SN neurons detect continuity of a preferred orientation, and SS neurons detect discontinuity of a preferred orientation.

## Materials and Methods

### Ethics statement

The experimental data were obtained from anesthetized and paralyzed adult cats (weighing 2.0∼4.0 kg each) bred in the facilities of the Institute of Neuroscience, Chinese Academy of Sciences (Permit Number: SYXK 2009-0066). This study was performed in strict accordance with the recommendations in the Guide for the Care and Use of Laboratory Animals from the National Institute of Health. The protocol was specifically approved by the Committee on the Ethics of Animal Experiments of the Shanghai Institute for Biological Sciences, Chinese Academy of Sciences (Permit Number: ER-SIBS-621001C). All surgeries were performed under general anesthesia combined with the local application of Lidocaine (for details see “animal preparation”), and all efforts were made to minimize suffering.

### Animal preparation

Acute experiments were performed on 10 cats. Detailed descriptions of the procedures for animal surgery, anesthesia, and recording techniques can be observed in a previous study [25]. Briefly, the cats were anesthetized prior to surgery with ketamine hydrochloride (30 mg/kg, intravenously [iv]), and then tracheal and venous canulations were performed. After surgery, the animal was placed in a stereotaxic frame for performing a craniotomy and neurophysiological procedures. During recording, anesthesia and paralysis were maintained with urethane (20 mg/kg/h) and gallamine triethiodide (10 mg/kg/h), respectively, and glucose (200 mg/kg/h) in Ringer's solution (3 mL/kg/h) was infused. Heart rate, electrocardiography, electroencephalography (EEG), end-expiratory CO_2_, and rectal temperature were monitored continuously. Anesthesia was considered sufficient when the EEG indicated a permanent sleep-like state. Reflexes, including cornea, eyelid, and withdrawal reflexes, were tested at appropriate intervals. Additional urethane was given immediately if necessary. The nictitating membranes were retracted, and the pupils were dilated. Artificial pupils of 3 mm diameter were used. Contact lenses and additional corrective lenses were applied to focus the retina on a screen during stimulus presentation. At the end of the experiment, the animal was sacrificed by an overdose of barbiturate administered i.v.

### Single-unit recording

The recordings were performed with a multi-electrode tungsten array consisting of 2
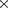
8 channels with 250 µm between neighboring channels from MicroProbes. The impedance of the electrodes was 5 MΩ on average (specified by the arrays' manufacture). The signals were recorded using the Cerebus system. Spike signals were band-pass filtered at 250–7500 Hz and sampled at 30 kHz. Only well-isolated cells satisfying the strict criteria for single-unit recordings (fixed shape of the action potential and the absence of spikes during the absolute refractory period) were collected for further analyses. We mapped and optimized the stimuli for each individual neuron independently and ignored the data simultaneously collected from other neurons. Recordings were made mainly from layers 2/3 and 4.

### Visual stimulation

The visual stimuli were generated by a Cambridge Systems VSG graphics board. The stimuli were patches of drifting sinusoidal gratings presented on a high-resolution monitor screen (40
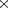
30 cm) at a 100 Hz vertical refresh rate. The screen was maintained at the identical mean luminance as the stimulus patches (10cd/m^2^).The monitor was placed 57 cm from the cat's eyes. All cells recorded were obtained from the area of the cortex that represented the central 10° of the visual field. When the single-cell action potentials were isolated, the preferred orientation, drifting direction, spatial frequency and temporal frequency of each cell were determined. Each cell was stimulated monocularly through the dominant eye with the non-dominant eye occluded.

To locate the center of the CRF, a narrow rectangular sine-wave grating patch (0.5°–1.0° wide
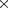
3.0°–5.0° long at a 40% contrast) was moved at successive positions along axes perpendicular or parallel to the optimal orientation of the cell, and the response to its drift was measured. The grating was set at the optimal orientation and spatial frequency and drifted in the preferred direction at the optimal speed for the recorded cells. The peak of the response profiles for both axes was defined as the center of the CRF.

We further confirmed that the stimulus was positioned in the center of the receptive field by performing an occlusion test, in which a mask consisting of a circular blank patch and concentric with the CRF, was gradually increased in size on a background drifting grating [15], [25], [26]. If the center of the CRF was accurately determined, the mask curve should begin at peak, and the response decreased as more of the receptive field was masked. If the curve obtained with the mask did not begin at the peak value, we considered the stimulus to be offset in relation to the receptive field center, and the position of the receptive field was reassessed.

In the size-tuning tests, the circular sinusoidal gratings (40% contrast) were centered over the receptive field center and randomly presented with different diameters (from 0.1 to 20°). The optimized values for these parameters (orientation, spatial and temporal frequency) were used in these tests. Each grating size was presented for 5–10 cycles of the grating drift, and standard errors were calculated for 3–10 repeats. We defined the CRF size as the aperture size of the peak response diameter (the stimulus diameter at which the response was maximal if the responses decreased at larger stimulus diameters or reached 95% of the peak value if they did not) [27], [28]. Contrast in the subsequent experiments was selected to elicit responses that reached approximately 90% of the saturation response for each cell with the center (CRF) contrast response function. Our contrast had a range of 20–70% and a mode of 40%.

To measure the orientation tuning of the cell's surround, the optimal orientation, aperture, and spatiotemporal frequencies for the center stimulus remained constant. Directly abutting the outer circumference of the center stimulus was a surround grating (with an outer diameter of 20°) of the identical phase and spatial and temporal frequencies. Whereas the center stimulus was maintained at the preferred orientation/direction throughout the experiment, the surround stimulus was shown with variable orientations/directions (in 15° increments). Both the center and surround stimuli were shown at a high contrast (with a range of 20–70% and a mode of 40%). The responses to each patch were recorded for 5–10 cycles of the grating drift, and standard errors were calculated for 3–10 repeats. The cells were classified as “simple” if the first harmonic (F1) of their response to the sine-wave gratings was greater than the mean firing rate (F0) of the response (F1/F0 ratio 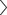
1)or “complex” if the F1/F0 ratio was 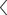
1 [29].

### Data analysis

The size-tuning curves for all recorded cells were fit using a DOG model [9]. In this model, the narrower positive Gaussian represented the excitatory center (the CRF), whereas the broader negative Gaussian represented the suppressive surround (the nCRF). The two Gaussians were considered to be concentrically overlapping, and the summation profile could be represented as the difference of the two Gaussian integrals. The model was defined by the following equation:
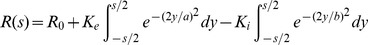
where *R*_0_ is the spontaneous firing rate, and each integral represents the relative contribution from putative excitatory and inhibitory components, respectively. The excitatory Gaussian is described by gain, *K*_e_, and a space constant, *α*, and the inhibitory Gaussian by its gain, *K*_i_, and space constant, *b*. All population values are provided below as the mean
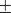
SEM.

## Results

The data were obtained from 119 neurons in the striate cortex of 10 cats. Of these neurons, 46 were simple cells and 73 were complex cells. The results did not differ significantly between these two classes.

### The determination of the extent of the classical receptive field and strength of surround suppression

**Figure 1:**
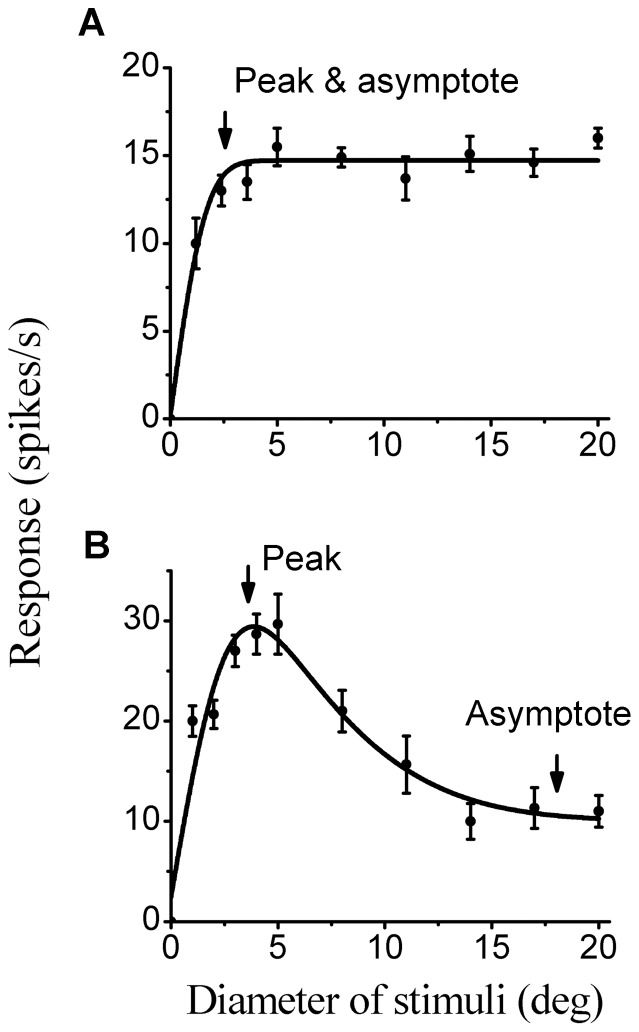
Size-tuning curves obtained from two representative cells. The response, in spikes per second (*y*-axis), is plotted against the diameter of the circular grating patch (*x*-axis). Solid lines indicate the best-fitting difference of integrals of the Gaussian functions. The arrows indicate stimulus diameter at which the responses were maximal and/or became asymptotic. (A) A cell exhibiting a no-suppression surround (SI = 0). (B) A cell showing a suppressive surround (SI = 0.65). The error bars represent
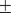
SEM.

The spatial extent of the classical receptive field (CRF) and the strength of surround suppression were determined using size-tuning tests (see materials and methods). Two patterns of behavior were distinguished based on the presence or absence of surround suppression. Fig. 1 shows the size-response curves of two representative cells. For some cells, the spike rate rose with increasing stimulus diameter and reached an asymptote (cell A). For other cells, the responses rose to a peak and then decreased as stimulus diameter further increased (cell B). The peak response diameter (as in Fig. 1B) or the diameter of the saturation point (reaching 95% of the peak value, as in Fig. 1A) was defined as the CRF size. Surround suppression was present for many of the cells tested, although its strength varied substantially between cells. We quantified the degree of surround suppression for each cell using the SI (1-asymptotic response/peak response). Across the population, suppression equal to or less than 10% was observed in 18.5% (22/119) of the cells tested, and these cells were assigned to the “surround-no-suppressive” category (SN). Cells that exhibited suppression greater than 10% were categorized as “surround-suppressive” (SS), which comprised 81.5% (97/119) of the population.

### The relationship between surround orientation selectivity and the degree of surround suppression

We initially determined the contrast-response function of the classical receptive field (CRF) for each individual neuron, and then a high-contrast that elicited a response of 90% of the saturation response (with a range of 20–70% and a mode of 40%) was selected to determine the neuron's preferred orientation by stimulating the CRF alone. We then varied the orientation of the surround grating and held the CRF stimuli in the cell's preferred orientation/direction. Altogether, we presented 24 surround orientations/directions shifted in 15° increments.

**Figure 2:**
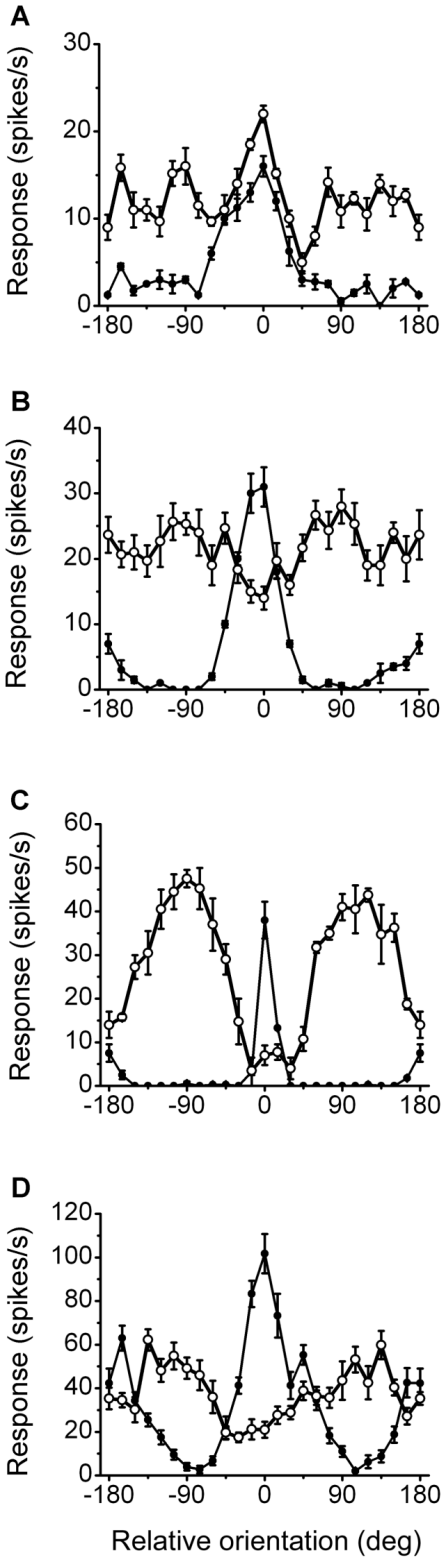
The orientation tuning curves of the center and surround for four representative neurons. The solid circles represent the orientation tuning of the center; 0° represents the optimal orientation for clarity. The open circles show the tuning of the surround when the center was stimulated with an optimally oriented grating. The error bars are
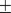
SEM. (A) The cell was identical to that shown in Fig. 1A, thus showing no surround suppression (SI = 0) in the size-tuning curve. A slight facilitation is shown when the surround grating was drifting in the preferred orientation/direction (0° on the *x*-axis) and a suppression drop occurred as the surround orientation was 45° away from the preferred orientation. (B) The neuron was identical to that in Fig. 1B, thus showing significant suppression (SI = 0.65) in the size-tuning test. The surround suppression was maximal in the optimal orientation/direction (0°) and weaker in the orthogonal orientations (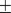
90° on the abscissa). (C) The neuron showed strong surround suppression around the optimal orientation/direction and significant facilitation in the orthogonal orientations. (D) The cell (SI = 0.67, size-tuning test) revealed a moderate-strength surround suppression to all surround orientations with a maximal suppression at −30°.

Figure 2 shows four representative examples of the surround orientation tuning curves when the central stimulus was in the preferred orientation/direction. The cells in Fig. 2 A and B were identical to those in Fig. 1 A and B. The solid circles plotted represent the center-alone condition, and the open circles plotted represent the orientation tuning measured by the surround grating when the center grating was optimal. The abscissa indicated the stimulus orientation relative to the neuron's preferred orientation/direction; 0 on the abscissa indicated that the gratings were oriented in the optimal orientation and drifted in the optimal direction. The cell in Fig. 2A possessed no surround suppression (SI = 0) in the size-tuning test (see Fig. 1A). A slight facilitation was shown on the orientation tuning curve when the surround grating was drifting in the neuron's preferred orientation/direction (0 position on the *x*-axis), and a suppression drop occurred when the surround orientation was 45° away from the preferred central grating. The cell illustrated in Fig. 2B displayed significant surround suppression (SI = 0.65) in the size-tuning test (see Fig. 1B) and on the surround orientation tuning curve. The surround suppression was strongest in the cell's optimal orientation/direction and weak in the orthogonal orientations (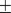
90° on the abscissa). The cell in Fig. 2C exhibited extremely strong suppression (SI = 0.98, size-tuning test) around the optimal orientation/direction (0 and 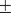
180°) and facilitation in the orthogonal orientations/directions. Fig. 2D shows a cell (SI = 0.67, size-tuning test) with moderate strength surround suppression at all surround orientations and a maximal suppression at −30°.

We consistently observed that the surround was more selective toward ortho-orientations compared to iso-orientations in the strong suppression neurons than in the weak suppression neurons. To quantify this observation, we used an index for iso-/orthogonal orientation selectivity (OSI). OSI is a measure of the average responses from 2 orthogonal surround orientations relative to the responses to the iso-oriented surround stimuli.
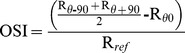
where R_θ0_ represents the response magnitude (spikes per second) at iso-orientation, R_θ−90_ and R_θ+90_ represent the response magnitude in the two orthogonal orientations, and R_ref_ is the response to the center stimulus presented alone.

OSI directly compared the suppression strength resulting from iso- and ortho-oriented surround stimuli. A value of 1 indicated that the surround had no suppressive effect in ortho-orientations and exerted complete suppression at iso-orientation, a value of 0 indicated that the suppression was equal in magnitude for both iso- and ortho-oriented surround stimuli, and negative values (OSI 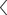
0) showed that the surround suppression was weaker in the iso- compared to ortho- orientation. The OSI values for the 4 cells in Fig. 2 A, B, C and D were −0.54, 0.41, 0.98 and 0.25, respectively.

**Figure 3:**
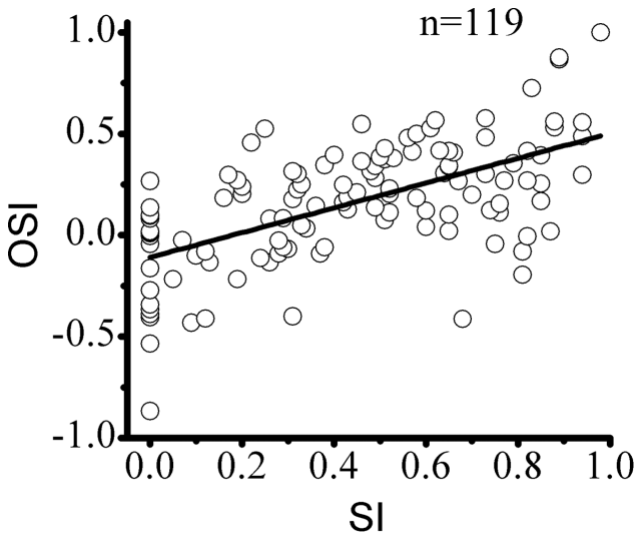
The relationship between the surround iso/orthogonal orientation selectivity index (OSI) and suppression index (SI). Each point represents data from one cell. The *x*-axis indicates the SI in the size-tuning tests, the *y*-axis indicates the surround iso-/orthogonal orientation selectivity index (OSI). The straight line is a linear regression of the data points. A significant correlation was observed between SI and OSI (r = 0.59, P
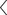
0.001).

The relationship between OSI and SI is shown in Fig. 3. The data revealed a significant positive correlation between the two variables (r = 0.59, P
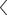
0.001). This result indicated that the stronger the surround suppression was, the greater the sensitivity to the ortho-orientation between the center and surround, whereas the weaker the surround suppression was, the greater the sensitivity to the iso-orientation between the center and surround.

**Figure 4:**
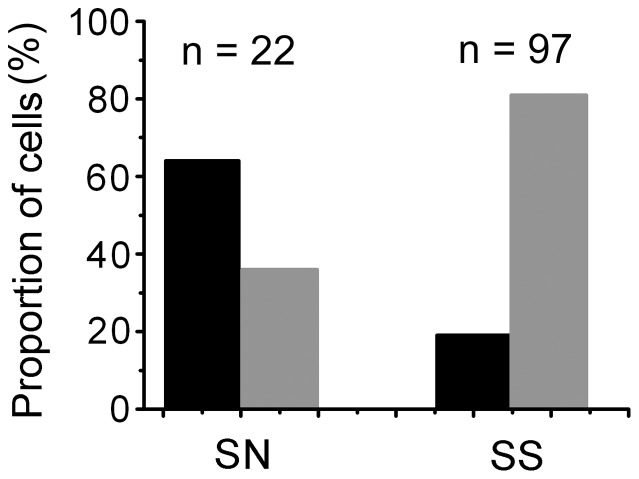
A comparison of OSI between the SN and SS neuron groups. The black columns indicate the proportion of cells with an OSI
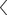
0, and the gray columns indicate the proportion of cells with an OSI
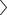
0. Within the 22 SN neurons, there were 14 cells (64%) with an OSI
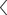
0 and 8 (36%) cells with an OSI
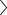
0. Comparing within the SS neuron group, the proportion was 19% (18/97) with an OSI
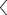
0 and 81% (79/97) with an OSI
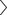
0.

**Figure 5:**
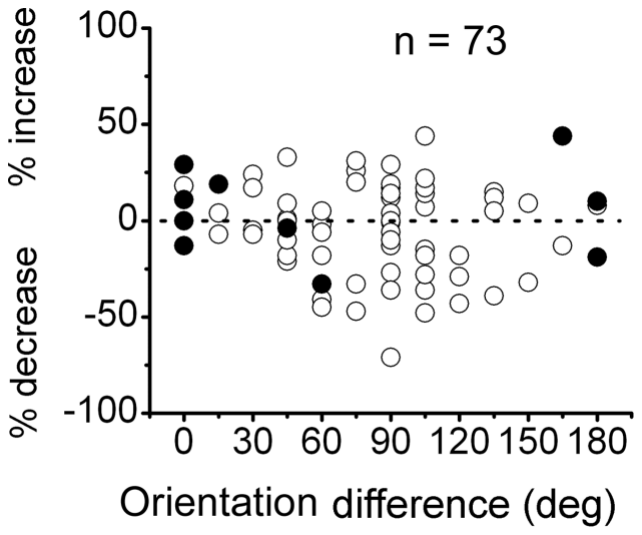
The distribution of differences between the preferred orientation of center and minimally suppressive orientation of the surround. The solid circles represent data from the SN cells (n = 11), and the open circles represent data from the SS cells (n = 62). The positions of the circles on the *x*-axis indicate the difference between the center optimal orientation and surround minimally suppressed orientation, and the position on the *y*-axis indicate the strength of the surround effect on the center response. The horizontal line represents response amplitude to the center stimulus alone, the circles underneath the line indicate the amplitude (in %) of the suppressive effect, and those above the line indicate the amplitude (in %) of the facilitative effect when the surround stimuli was oriented in the minimally suppressive orientation.

Of the 119 neurons analyzed, there were 22 SN cells and 97 SS cells. The distribution of OSI within the two groups is shown in Fig. 4. The black columns indicate the proportion of cells with an OSI
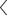
0, and the gray columns indicate the proportion of cells with an OSI
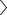
0. Within the 22 SN neurons, there were 14 cells (64%) with an OSI
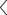
0 and 8 (36%) cells with an OSI
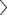
0. A comparison within the SS neuron group indicated that the proportion was 19% (18/97) with an OSI
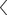
0 and 81% (79/97) with an OSI
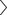
0. Because SN neurons exert weaker suppression to iso-oriented surround stimuli and vice versa for the SS cells, these results implied that the SN neurons were more suitable for detecting orientation similarity or continuity, whereas the SS neurons detected differences or discontinuity in orientation. The relationship between the preferred orientations of the center and minimally suppressive orientation of the surround for the two types of neurons (SN and SS groups) is compared in Fig. 5. We included only those cells in the sample that showed a statistically significant peak in the surround tuning curve. The solid circles represent the SN cells (n = 11), and the open circles represent the SS cells (n = 62). The positions of the circles on the *x*-axis indicate the difference between the center optimal orientation and minimally suppressed orientation of the surround, and the positions on the *y*-axis indicate the strength of the surround effect on the center response. The horizontal line represents the response amplitude to the center stimulus alone. The circles underneath the line indicate suppressive effect, and those above the line indicate facilitative effect, whereas the surround stimuli were oriented in the minimally suppressive orientation. Although the positions of the minimally suppressive orientation varied between cells, the maxima of the SS cells tended to aggregate around the orthogonal or oblique orientation, and for the maxima of SN cells, the cells tended to aggregate around the preferred orientation of center (CRF). The remaining cells (46/119) showed a broadly tuned surround or were nearly uniformly suppressive at all orientations.

### The population response of the two types of neurons

**Figure 6:**
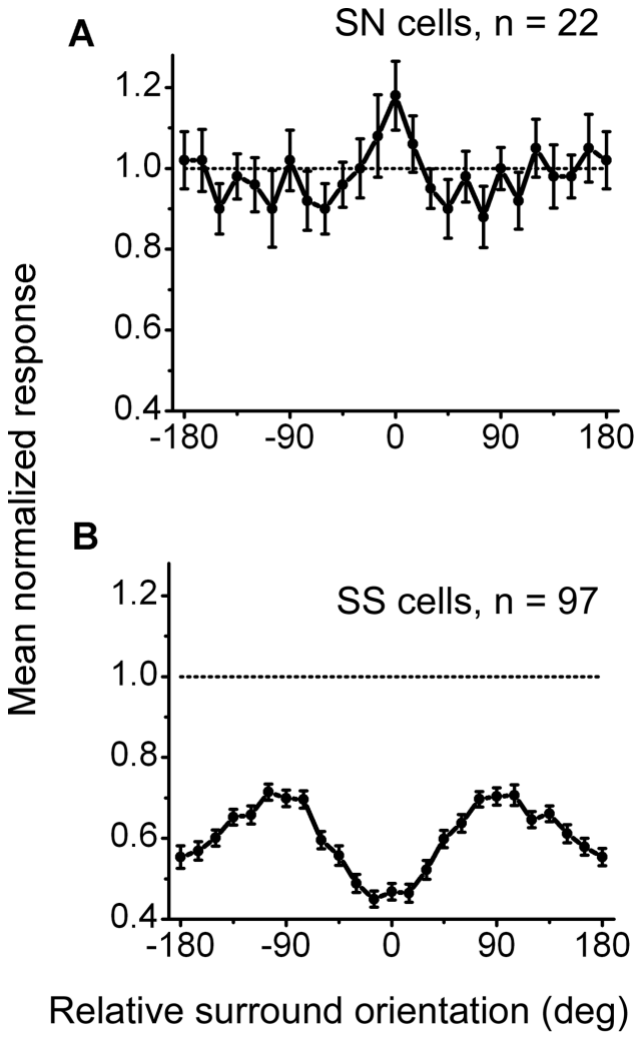
A population analysis of the orientation/direction-dependent surround effects of the SN and SS neurons. The surround response amplitude of each neuron was normalized relative to the center response. The horizontal line indicates the response to the preferred center stimulus alone. (A) The averaged surround tuning curve of 22 SN cells shows that the surround stimuli have no significant effects at most orientations but display significant facilitation in the neuron's preferred orientation/direction. (B) The averaged surround tuning curve of 97 SS cells. The neurons were maximally suppressed by surround stimuli in the neuron's preferred orientation/direction and were least suppressive in the orthogonal orientation/direction (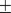
90°).

To assess the overall effects of surround stimulation on the center response, we pooled the data individually for the two categories of cells by normalizing the magnitude of the surround effect to center stimulation alone. Figures 6A and B show the mean surround orientation tuning curves for the SN and SS cells, respectively. Fig. 6A shows that the SN neurons (n = 22) produced no significant effects from the surround stimulation at most orientations but significant facilitation in the neuron's preferred orientation/direction. Fig. 6B shows that the SS neurons (n = 97) were suppressed by surround stimuli at all orientations with the strongest suppression (minimum response) in the neuron's preferred orientation and direction.

## Discussion

Previous studies [10], [30] have shown that V1 neurons can detect orientation discontinuity between CRF and its surround as these neurons respond to any combination of center-surround orientations as long as the two orientations are not identical. Other studies [7], [9], [11], [15], [19], [31]–[34] have reported that the response tended to be stronger when the orientations of the surround stimuli were orthogonal to the optimal orientation of the center.

Levitt and Lund [11] reported that for some neurons, the sensitivity to the surround orthogonal orientation became weaker by using a lower contrast of the central stimulus. A recent study [24] reported that the surround orientation tuning could be related to the position of the cells in the optical orientation map, and the authors observed that orthogonal orientation selectivity was higher in the domain cells and lower in the pinwheel cells.

In the present experiments, we recorded 119 neurons from the V1 of cats using high-contrast stimuli. According to the presence or absence of a suppressive surround in the size-tuning tests, we divided these neurons into two categories: surround-suppressive (SS) and surround-non-suppressive (SN). Of the sample we recorded, 82% (97/119) were SS cells with a significant suppressive surround, and 18% (22/119) were SN cells with no suppression or slight facilitation over the surround. We analyzed the relative orientation selectivity between the center and surround using the iso/orthogonal orientation selectivity index (OSI), and observed that the OSI of the surround depended significantly on the suppressive strength (*SI*) of the neurons. The stronger the surround suppression was, the greater the sensitivity was to the orthogonal orientation between the center and surround, whereas the weaker the surround suppression was, the greater the sensitivity was to the iso-orientation between the center and surround. When both the center and surround were stimulated by the optimal orientation of the cells, the response of the SS cells were maximally suppressed, whereas the response of the SN cells were activated or facilitated. From these results, it can be concluded that SS cells are possibly suitable for the detection of differences or discontinuity in orientations (ortho- or oblique- orientation between the surround and optimal center), and conversely, SN cells are most likely adapted to detecting similarities or continuity of orientations (the identical preferred orientation and drift direction between the surround and center).

The functionally different iso/orthogonal center-surround interactions in orientation selectivity might be based on distinct local cortical circuitry for the two types of neurons. Earlier studies reported that some pyramidal cells in the cat visual cortex exhibit extensively spreading horizontal axon collaterals that link neighboring regions over several millimeters [35]–[43]. In our previous paper [44], we compared the morphological features of the suppressive and non-suppressive (or facilitative) neurons in the primary visual cortex of anesthetized cats (Fig. 1 and Fig. 2 in [44]). We observed that only the non-suppressive (or facilitative) neurons possessed such extensively spreading axon collaterals, whereas the strong-suppressive neurons had restricted local axons. The SN neurons most likely detect orientation continuity by using their long-distance horizontal connections, which provide connections between neighboring cells with similar orientation preferences [37], [38], [41], [45]–[47], whereas the strong suppression neurons detect orientation discrepancy using local suppressive connections [48].

## Conclusions

Our results demonstrate that 1) the surround-non-suppressive (SN) neurons tended to detect center-surround continuing orientation and surround-suppressive (SS) neurons possibly to select center-surround discontinuing orientation, and 2) the magnitude of the iso-/orthogonal orientation sensitivity of the surround significantly depended on the suppressive SI of the neurons.
